# Expression of evolutionarily novel genes in tumors

**DOI:** 10.1186/s13027-016-0077-6

**Published:** 2016-07-19

**Authors:** A. P. Kozlov

**Affiliations:** The Biomedical Center and Peter the Great St. Petersburg Polytechnic University, St. Petersburg, Russia

## Abstract

The evolutionarily novel genes originated through different molecular mechanisms are expressed in tumors. Sometimes the expression of evolutionarily novel genes in tumors is highly specific. Moreover positive selection of many human tumor-related genes in primate lineage suggests their involvement in the origin of new functions beneficial to organisms.

It is suggested to consider the expression of evolutionarily young or novel genes in tumors as a new biological phenomenon, a phenomenon of *TSEEN* (tumor specifically expressed, evolutionarily novel) genes.

## Background

Evolutionarily novel genes are those novel genes which originate in the germ cells of multicellular organisms and thus can participate in evolution. Genes that originate in somatic cells (e.g. in tumor cells) and cannot be passed to the progeny organisms are not considered as evolutionarily novel.

Novel genes can originate from pre-existing genes or *de novo*. The theory of the origin of novel genes is well developed and the mechanisms of the origin of evolutionarily novel genes are well understood and described [8, 45, 58, 70, 76, 77, 110, 131, 132, 189, 194, 217]. But there is a question in which cells of the evolving multicellular organisms genes determining the evolutionary innovations and morphological novelties are expressed.

There is a general correlation between the increase in the gene number in the genomes of evolving organisms, from one side, and the increase in the number of cell types, the origin of other innovations and the overall complexity, on the other [34, 91, 215]. The question is how such adequate correlation was realized at the multicellular level. An adequate increase in cell number that accompanied the process of the origin of novel genes is hard to imagine. More likely, some autonomous cellular proliferative processes were recruited to provide the space for the expression of new genes.

In my previous publications [88–90] and in my recently published book “Evolution by Tumor Neofunctionalization” [91] I suggested that heritable tumors – benign tumors or tumors at the early stages of progression – may provide extra cell masses for expression of evolutionary novel genes and for emergence of evolutionary innovations and morphological novelties. The non-trivial prediction of this hypothesis is that we may find the expression of evolutionarily novel genes in tumors.

Experiments in this direction performed in my lab since early 2000s have indeed demonstrated the specific or predominant expression of many evolutionarily young or novel genes in tumors. These data will be discussed in the first part of this review.

I also found in the literature descriptions of many genes with similar dual specificity – tumor specifically expressed, evolutionary novel. Such genes with dual specificity were not purposefully searched for by the authors and the connection of tumors and evolution was not emphasized. Rather, the data on evolutionary novelty and specificity of expression of certain genes were the result of descriptive experiments and often can be found among other described features of the studied genes. Similar information may be found in the results of genome-wide studies. Tumor specificity of expression of genes originated by gene duplication, from retrotransposons and endogenous retroviruses, by exon shuffling or *de novo* will be discussed in the second part of this review.

### The purposeful experimental search for evolutionarily novel genes with tumor-specific expression

To study experimentally the prediction concerning the expression of evolutionarily young or novel genes in tumors we used two complementary approaches. One was to study the evolutionary novelty of genes/sequences with proven tumor specificity of expression. The other was to study tumor specificity of expression of genes/sequences with proven evolutionary novelty. Both approaches found out genes/sequences with dual specificity, i.e. tumor-specifically or tumor-predominantly expressed and evolutionarily young or novel.

#### The evolutionary novelty of tumor-specifically expressed sequences

To find the sequences which are expressed in tumors but not in normal tissues the global comparison of cDNA sequences from all available tumor-derived libraries with cDNA sequences from all available normal tissue-derived libraries was performed. The normal EST set was subtracted *in silico* from the tumorous EST set [11].

The results showed that, in accordance with my prediction, tumors indeed express hundreds of sequences that are not expressed in normal tissues. About half of discovered tumor-specific sequences lack long reading frames (i.e., may be referred to non-coding RNAs) and defined function [11, 51]. Among non-coding RNAs, the long non-coding RNA [94] and candidate microRNA (see ELFN1-AS1, a novel primate gene expressed predominantly in tumors) have been described.

The analysis of the relative evolutionary novelty of sequences retrieved from the paper [51] was performed. The protein-coding sequences were studied by ProteinHistorian tool [28]. The nucleotide BLAST algorithm and the original Python script [3] were used to analyze the novelty of noncoding sequences. The orthologs of tumor-specifically expressed sequences described by Baranova and co-authors were searched in 26 completely sequenced eukaryotic and prokaryotic genomes. The curves of phylogenetic distribution of orthologs of these sequences have been generated. The data suggest that both sets of tumor-specifically expressed sequences are relatively evolutionary novel. The non-coding tumor-specifically expressed sequences are younger than protein-coding tumor-specifically expressed sequences. During last 39 million years of evolution, these sequences represented the youngest gene class in human ancestors’ genomes [115, 116].

In vitro experiments intended to confirm that the sequences found *in silico* are indeed specifically expressed in tumors were also carried out. cDNA panels from normal and tumor tissues were used for PCR with specific primers. In total, 56 sequences described in [11] have been studied in this way. Among them, nine were confirmed to be highly tumor-specific [94, 95, 138]. The sequences that have been confirmed to be tumor-specific are expressed in a vast variety of tumors. For example, the sequence Hs. 202247 is expressed in 46 tumor samples out of 56 examined and in none of 27 normal tissues. One of the protein products of the sequences that proved to be tumor-specific appeared to be a promising immunogen for antitumor vaccine development [138, 170]. However, most of experimentally confirmed tumor-specific sequences appear to be non-coding RNAs.

The nine experimentally confirmed tumor-specific sequences were studied for their evolutionary novelty using molecular-biological techniques, comparative genomics analysis, the search for orthologous sequences and sequence conservation analysis [92, 163, 164]. Eight of the nine tumor-specifically expressed sequences are either evolutionarily new (primates or humans) or relatively young (mammals) (Table 1) and evolve neutrally [92, 93, 162–164]. I suggest to call such sequences Tumor-Specifically Expressed, Evolutionarily New Sequences, or *TSEEN* sequences.

**Table 1:** Evolutionarily novel and young genes with tumor specific or predominant expression studied at the Biomedical Center

Single genes obtained by *in silico* subtraction studied for evolutionary novelty					
Gene	Protein/RNA	Expression in tumors	Expression in normal tissues	Evolutionary novelty ^a^	References
Orthopedia homeobox (*OTP*) Hs.202247	RNA	Lung, colon, ovary, stomach, breast, kidney, bladder, uterus, lymphomas, testis, small intestine, esophagus	No	Mammalia	[11, 91–95, 162–164]
Embryonic stem cell related (non-protein coding) (*ESRG*) (Hs.720658)	RNA	Ovary, testis, lung, lymphomas	PBL, testis, thymus	Humans	[11, 91–95, 162–164]
Transcribed locus chr.8 (q24.21) (Hs.666899)	RNA	Lung, colon, prostate, kidney, bladder, uterus, breast, testis, lymphomas, ovary, stomach	Lymph node, spleen (very weak), thymus (very weak)	Mammalia	[11, 91–95, 162–164]
Transcribed locus chr.7 (q21.13) (Hs.150166)	RNA	Esophagus, stomach, small intestine, kidney, bladder, uterus, ovary, breast, lung, testis, lymphomas	Heart (very weak), kidney (very weak), lung (very weak)	Mammalia	[11, 91–95, 162–164]
* ELFN1* antisense RNA 1 (non-protein coding) (Hs.633957)	RNA	Lung, colon, prostate, ovary, breast, uterus, testis, lymphomas, esophagus, stomach, small intestine	Liver, heart (very weak), stomach (very weak)	Primates	[11, 91–95, 149–152, 162–164]
Intergenic spacer region within rRNA repeating unit (Hs.426704)	RNA	Breast, pancreas, esophagus, liver, small intestine, testis, lung	No	Primates	[11, 91–95, 162–164]
HERV-H LTR-associating 1 (*HHLA1*) (Hs.285026)	Protein ?	Esophagus, stomach, small intestine, bladder, uterus, breast, testis	Bone marrow (very weak)	Humans	[11, 91–95, 162–164]
Small Proline-Rich Protein 1A (*SPRR1A*) (Hs.46320)	Protein	Lung, esophagus, colon, bladder, testis	Thymus (very weak)	Mammalia	[11, 91–95, 162–164]
Evolutionarily novel single genes studied for tumor specificity of expression					
Gene	Protein/RNA	Evolutionary novelty	Expression in tumors	Expression in normal tissues	References
Chronic lymphocytic leukemia up-regulated1 (*CLLU1*) (Hs.730377)	Protein	Humans	Lung, stomach, prostate, spleen	No	[97]
Dermicidin (*DCD*) (Hs.350570)	Protein	Primates	Breast, kidney, skin, skeletal muscle, parotid	Thymus, PBL (very weak), testis (very weak), lymph node (very weak)	[97]
Prostate and breast cancer overexpressed 1 (*PBOV1*) (Hs.302016)	Protein	Humans	Brain, lung, liver, gallbladder, stomach, small intestine, colon, breast, uterus, ovary, cervix, ureter, bladder, prostate, testis, adrenal, parotid, thymus, spleen, lymphomas	No	[96, 165]
Tumor- related gene classes studied for evolutionary novelty					
Class of genes		Number of genes	Evolutionary novelty		References
CT –antigen genes		276	36.7 % of human CT-genes originated in Catarrhini, Hominidae and humans		[44]
			30 %- in Eutheria		
CT-X-antigen genes		60	31.4 % of CT-X genes are exclusive to humans		[44]
			39.1 % have ortologs in Catarrhini and Homininae		
BMC globally subtracted, tumor-specifically expressed non-coding sequences		110	30 % originated after Catarrhini		[51, 115, 116]
			16 % originated after Homininae		
BMC globally subtracted, tumor-specifically expressed protein-coding sequences		73	More than 30 % originated after Eutheria		[51, 115, 116]

^a^The use of the different tool results in six “primates”, one “humans” and one “mammalia” in this column (Makashov, personal communication)

The sequence Hs.285026 (*HHLA1*) contains ORF, although the corresponding protein is not shown experimentally. This sequence is similar to human *de novo* protein-coding genes [86]. As far as corresponding protein has not been shown, this sequence may represent the earlier stage of the novel gene origin comparing to those described by D.G. Knowles and A. McLysaght. This and other sequences described in our studies (besides protein-coding sequences with established functions) may represent proto-genes (gene precursors which have not yet acquired functions and evolve neutrally [29]) at different stages of their evolution towards novel genes with protein or RNA related functions. The sequence Hs.633957 represents this transition.

#### *ELFN1-AS1*, a novel primate gene expressed predominantly in tumors

The human transcribed locus resides in the 7th chromosome and corresponds to the UniGene EST cluster Hs.633957. It was found by our group to be expressed in a tumor-specific manner by *in silico* analysis [11]. Later these data were supported experimentally: specific transcripts of the locus were detected in tumors of various histological origins, but not in most of the healthy tissues [94, 149, 150].

Experimental and *in silico* evidence that locus is a stand-alone gene which has its own promoter and capability for alternative splicing was obtained. However, only one splicing isoform is predominant. The gene was assigned a gene symbol *ELFN1-AS1*, *ELFN1* antisense RNA 1 (non-protein coding), gene name approved by Human Gene Nomenclature Committee. Our data point to the miRNA function of *ELFN1-AS1* with DPYS mRNA being its primary target [151, 152].

This gene originated *de novo* from an intronic region of a conservative gene *ELFN1* (NCBI Ref. Seq. NM_001128636.2) in primate lineage. Homologous sequences of this gene were identified by us in all primates, but the DNA sequence from the representative of suborder Strepsirrhini *Otolemur garnettii* has more than 50 % differences from its human counterpart and forms an outgroup on the phylogenetic tree. Thus *ELFN1-AS1* could become transcriptionally active after divergence of Strepsirrhini and Haplorhini primates. It is noteworthy that all the Haplorhini primates have a region with 5 or more E-boxes downstream of the DS site. This suggests that *ELFN1-AS1* gene since its origin could be c-Myc-responsive.

Taken together, the data indicate that human transcribed locus contains a gene for some non-coding RNA, likely a microRNA. This gene combines features of predominant expression in tumors and evolutionary novelty [151, 152].

#### *PBOV1, de novo* originated human gene with tumor-specific expression

In the study of *PBOV1* gene the other approach was used, i.e. the evolutionary novelty of the gene was studied first.

*PBOV1* (*UROC28*, *UC28*) is a human protein-coding gene with a 2501 bp single-exon mRNA and 135aa ORF. The gene has been originally characterized by An and co-workers [4]. This gene was mentioned among 12 human genes without orthologs in the mouse and dog genomes in the paper of Clamp and co-authors [38]. We studied the evolutionary novelty of this gene more carefully and found that the coding sequence of *PBOV1* is poorly conserved in the mammalian evolution and originated *de novo* in primate evolution through a series of frame-shift and stop codon mutations. Consequently, 80 % of protein sequence is unique to humans. The Ka/Ks ratio both in pairwise alignments and in multiple alignment of all primate sequences syntenic to human coding sequence didn’t show any significant differences from 1.0, indicating that the amino acid sequence evolved neutrally. PBOV1 protein lacks any annotated or predicted domains and over 60 % of its sequence is predicted to be disordered. These findings strongly suggest that human PBOV1 is a protein of a very recent *de novo* evolutionary origin [165].

**Fig. 1:**
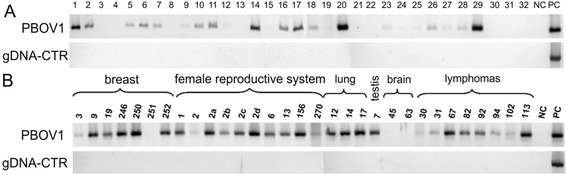
PBOV1 expression measured by PCR in cDNA panels from human tumors. **a** Tumor cDNA Panel (BioChain Institute, USA): 1 – Brain medulloblastoma, with glioma, 2 – Lung squamous cell carcinoma, 3 – Kidney granular cell carcinoma, 4 – Kidney clear cell carcinoma, 5 – Liver cholangiocellular carcinoma, 6 – Hepatocellular carcinoma, 7 – Gallbladder adenocarcinoma, 8 – Esophagus squamous cell carcinoma, 9 – Stomach signet ring cell carcinoma, 10 – Small Intestine adenocarcinoma, 11 – Colon papillary adenocarcinoma, 12 – Rectum adenocarcinoma, 13 – Breast fibroadenoma, 14 – Ovary serous cystoadenocarcinoma, 15 – Fallopian tube medullary carcinoma, 16 – Uterus adenocarcinoma, 17 – Ureter papillary transitional cell carcinoma, 18 – Bladder transitional cell carcinoma, 19 – Testis seminoma, 20 – Prostate adenocarcinoma, 21 – Malignant melanoma, 22 – Skeletal Muscle malignancy fibrous histocytoma, 23 – Adrenal pheochromocytoma, 24 – Non-Hodgkin’s lymphoma, 25 – Thyroid papillary adenocarcinoma, 26 – Parotid mixed tumor, 27 – Pancreas adenocarcinoma, 28 – Thymus seminoma, 29 – Spleen serous adenocarcinoma, 30 – Hodgkin’s lymphoma, 31 – T cell Hodgkin’s lymphoma, 32 – Malignant lymphoma. NC – PCR with no template, PC – PCR with human DNA. **b** PBOV1 expression in clinical tumor samples. PBOV1 is expressed in breast cancer (9–250), ovary cancer (1, 6), cervical cancer (2, 13), endometrial cancer (156, 270), lung cancer (12, 14, 17), seminoma (7), meningioma (63), non-Hodgkin lymphomas (67, 82, 92, 102, 113). From open access paper [165]. Copyright of authors

**Fig. 2:**
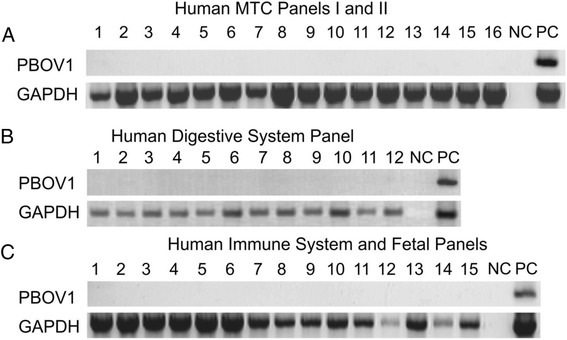
Expression of PBOV1 and GAPDH (positive control) measured by PCR in cDNA panels from human normal tissues. **a** Human MTC Panel I (1–8), Human MTC Panel II (9–16): 1 – brain, 2 ¬– heart, 3 – kidney, 4 – liver, 5 – lung, 6 – pancreas, 7 – placenta, 8 – skeletal muscle, 9 – colon, 10 – ovary, 11 – peripheral blood leukocyte, 12 – prostate, 13 – small intestine, 14 – spleen, 15 – testis, 16 – thymus. **b** Human Digestive System MTC Panel: 1 – cecum, 2 – colon, ascending 3 – colon, descending 4 – colon, transverse 5 – duodenum, 6 – esophagus, 7 – ileocecum, 8 – ileum, 9 – jejunum, 10 – liver, 11 – rectum, 12 – stomach. **c** Human Immune System MTC Panel (1–7), Human Fetal MTC Panel(8–15): 1 – bone marrow, 2 – fetal liver, 3 – lymph node, 4 – peripheral blood leukocyte, 5 – spleen, 6 – thymus, 7 – tonsil, 8 – fetal brain, 9 – fetal heart, 10 – fetal kidney, 11 – fetal liver, 12 – fetal lung, 13 – fetal skeletal muscle, 14 – fetal spleen, 15 – fetal thymus; A-C: NC – PCR with no template, PC – PCR with human DNA. From open access paper [165]. Copyright of authors

After establishing the evolutionary novelty of *PBOV1* gene, the specificity of its expression in tumors and normal tissues was studied. *PBOV1* has been previously reported to be overexpressed in prostate, breast, and bladder cancers [4]. We studied the expression of *PBOV1* using PCR on panels of cDNA from various normal and tumor tissues. The gene had a highly tumor-specific expression profile. It was expressed in 20 out of 34 tumors of various origins but was not expressed in any of the normal adult or fetal human tissues that we tested (Figs. 1 and 2). The interesting feature of this result is that tumor specificity of *PBOV1* expression was predicted by us from its evolutionary novelty [96, 165].

Unlike cancer/testis antigens genes *PBOV1* is expressed from a GC-poor TATA-containing promoter which is not influenced by DNA methylation and is not active in testis. *PBOV1* activation in tumors may depend on sex hormone receptors, C/EBP transcription factors and Hedgehog signaling pathway. Although the PBOV1 protein has recently originated *de novo* and thus has no identifiable structural or functional signatures, a missense SNP (single nucleotide polymorphism) in it has been previously associated with an increased risk of breast cancer. Using publicly available data we found that higher level of *PBOV1* expression in breast cancer and glioma samples were significantly associated with a positive disease outcome. *PBOV1* is also highly expressed in primary but not recurrent high-grade gliomas, suggesting that immunoediting against *PBOV1*-expressing cancer cells might occur over the course of disease. We propose that *PBOV1* is a novel tumor suppressor gene which might act by provoking the cytotoxic immune response against cancer cells that express it. We speculate that this property might be a source of phenotypic feedback that facilitated *PBOV1* gene fixation in human evolution [165].

#### The evolutionary novelty of human cancer/testis antigen genes

Cancer/testis antigen genes (CTA or CT genes) code for a subgroup of tumor antigens expressed predominantly in testis and different tumors. CT antigens may be also expressed in placenta, in female germ cells, and in the brain [33, 64, 175, 209, 210] (see discussion of CT genes expression in the brain in [91]). At the time of the study, CTDatabase (http://www.cta.lncc.br) included 265 CT genes and 149 CT gene families.

The hypothesis of the expression of evolutionarily novel genes in tumors explains this otherwise strange cancer-testis association paradox: as far as the origin of evolutionarily novel genes is connected with their expression in germ cells, cancer/testis genes are novel genes which are expressed in tumors.

So I suggested that cancer/testis antigen genes should be evolutionarily new or young genes. In order to prove this prediction, the presence of genes orthologous to human cancer-testis genes in human lineage was studied [44]. This analysis was performed separately for genes located on the X chromosome and autosomal cancer/testis genes, as far as extensive traffic of novel genes has been described for mammalian X chromosome [16, 46, 103].

Orthologs of each of CT genes were searched among annotated genes in several completely sequenced eukaryotic genomes using HomoloGene tool of NCBI [168] and distributions of orthologs of all CT-X genes, all autosomal CT genes, all human CT genes and all annotated protein coding genes from human genome in 11 taxa of human evolutionary lineage were built. It was shown that 31.4 % of CT-X genes are exclusive for humans and 39.1 % of CT-X genes have orthologs originated in Catarrhini and Homininae. Thereby the majority of human CT-X genes (70.5 %) are novel or young for humans.

Altogether 36.7 % of all human CT genes originated in Catarrhini, Homininae and humans. It was also found that 30 % of all human CT genes originated in Eutheria. These CT genes acquired functions in Eutheria. This indicates the importance of processes in which tumors and CT antigens were involved during the evolution of Eutheria. CT genes originated in Eutheria are located mainly on autosomes. CT genes originated in Catarrhini, Homininae and humans are located predominantly on X chromosome. This difference is probably related to important events in evolution of mammalian X chromosome since the origin of Eutheria [99], especially to the acquisition of a special role in the origin of novel genes [77].

Thus the majority of CT-X genes are either novel or young for humans, and majority of all human CT genes (>70 %) originated during or after the origin of Eutheria. These results suggest that the whole class of human CT genes is relatively evolutionarily new [44].

Our data are in good correspondence with evidence obtained by other groups on particular families of CT genes. I found the evidence in the literature that at least 7 families (of 149 families know by that time) of CT genes (*MAGE-1, PRAME, SPANX-A/D, GAGE, XAGE, CT45* and *CT47*) and many CT genes located on the X chromosome (CT-X genes) were either new or young (reviewed in [91]. Later it was found that one more CT gene family, *CTAGE* (cutaneous T-cell-lymphoma-associated antigen) shows a rapid and primate specific expansion, especially in humans, which starts with an ancestral retroposition in the Haplorhini ancestor followed by DNA-based duplications [214]. But our study [44] was the first systematic study of the evolutionary novelty of the whole class of CT genes which showed that it is relatively evolutionarily novel. Thus our prediction of the evolutionary novelty of the whole class of CT genes turned out to be correct.

The relative evolutionary novelty of the whole class of CT genes confirms the prediction about expression of evolutionarily young and novel genes in tumors. The expression of cancer/testis genes in tumors thus appears as a natural phenomenon, not an aberrant process as interpreted by most of authors (e.g. [1, 27, 32, 36, 175, 214]). More discussion of evolutionary novelty of CT genes may be found in my recent book [91].

The list of single genes and gene classes studied by our group at the Biomedical Center is presented in Table 1.

The data obtained by our group, both on individual genes and on large groups of genes, suggest that tumor specifically expressed, evolutionarily novel (*TSEEN*) genes could represent a new biological phenomenon, a phenomenon of *TSEEN* genes [91]. That is why I looked in the literature for the evidence about similar kind of genes, i.e. evolutionarily novel, tumor specifically expressed.

### Analysis of the literature data related to *TSEEN* genes

It turned out that many examples of genes with dual specificity –evolutionarily novel, tumor specifically expressed – could be found in the literature but serious attention was never paid to this association. Below I will discuss the tumor specificity of expression of genes originated by different mechanisms - by gene duplication, from retrotransposons and endogenous retroviruses, by exon shuffling or *de novo*. As far as positive Darwinian selection is a feature of many evolutionarily novel genes, human tumor-related genes positively selected in primate lineage will be also discussed.

#### Expression of pseudogenes in tumors

Gene duplication is a major way of genome evolution. The original hypothesis [131] suggested that pre-existing genes are under control of natural selection, and their evolution is constrained within their existing function. The extra copy of existing gene gets out of control of the natural selection, so that accumulation of mutations in this extra copy may lead to the origin of a novel gene with related or even new function. Gene duplication is considered as providing the “row material” for the origin of new genes. This concept also suggests that the majority of duplicates becomes inactive pseudogenes due to degenerative mutations, and only rarely beneficial mutations would lead to the emergence of a new gene with a novel function [131]. But the term “pseudogene” was first introduced by C. Jacq and co-authors in 1977 [72].

The DNA-mediated mechanisms of gene duplication include unequal crossing over, tandem, segmental, chromosomal or genome duplications. The resulting gene duplicates may be organized in tandem, interspersed or polyploid manner. Segmental duplications are large interspersed segments of DNA with high sequence identity (>90 %), usually separated by >1Mb of unique sequences [120].

RNA-based gene duplication, or retroposition, creates duplicate genes by reverse transcription of RNAs from parental genes. RNAs from all categories generate retrosequences that may be exapted as novel genes or regulatory elements [21]. Retrogenes are most abundant in mammals where long interspersed nuclear elements (LINEs) that provide the enzyme reverse transcriptase for retroposition are widespread. The majority of retrogenes is produced by genes with high levels of germline expression. They often originate from the X chromosome [16, 76]. A new retrogene is intronless, contains a poly(A) tract, and may be flanked by short duplicate sequences [15, 104].

DNA-mediated gene duplication is more frequent event in genome evolution, while RNA-based gene duplication is more capable to generate genes with novel functions. The retroposition is less likely to provide expressed daughter retrocopies than segmental DNA duplication because retrocopies do not contain regulatory elements. So, new promoters and enhancers should somehow be recruited for the origin of new genes, and several mechanisms of such recruitment are described [76, 77]. Retrogenes usually locate on chromosomes different from that of parental genes. Mammalian X chromosome demonstrates extensive retrogene traffic [46]. For reasons of different location and new promoter recruitment, the transcribed retrogenes are more capable to evolve new expression patterns and novel functional roles than gene duplicates arising by DNA segmental duplication [76, 77]. Retrogenes, like duplicates originated through DNA-mediated mechanisms, might provide the raw material for the origin of evolutionarily novel genes and functionally important evolutionary innovations [76, 119, 197]. At least one functional retrogene per million years originated in primate lineage that led to humans [119].

In accordance with two major ways of gene duplication – DNA-based and RNA-based mechanisms – two types of pseudogenes are categorized as duplicated and processed pseudogenes, accordingly [105, 148]. One more group of pseudogenes includes so called “unitary” pseudogenes that arise through spontaneous mutations of single coding genes [216]. Other pseudogene biotypes may include polymorphic pseudogenes (loci known to be coding in some individuals), IG pseudogenes (immunoglobulin segments with disabling mutations) and TR pseudogenes (T-cell receptor gene segments with disabling mutations) [147].

Hundreds to thousands of pseudogenes have been identified in different species. In humans, 11,216 pseudogenes have been recently annotated, including ~8,000 processed pseudogenes [61, 147]. The extrapolation estimates suggest that the number of pseudogenes in human genome may be ~14,000 [147]. This is smaller than earlier estimates [190, 217]. The processed pseudogenes are the most abundant type of pseudogenes in human genome which is connected with the burst of retroposition activity in ancestral primates [135, 217]. Pseudogenes have long been considered as non-functional or “junk” DNA. But during the last decade, the attitude has changed substantially. The evidence is accumulating that many pseudogenes are transcribed and functional in development and diseases (reviewed in [105, 148, 154, 173]. Laura Poliseno determines the following types of pseudogene functions: related to the parental gene and parental gene independent functions; mediated by the pseudogene DNA, by pseudogene RNA transcribed in sense, by pseudogene RNA transcribed in antisense, or by pseudogene-encoded proteins [154]. Pseudogenes transcribed as noncoding RNAs may regulate their parental genes as antisense RNAs, short interfering RNAs (siRNAs) or as microRNA decoys [173]. Pseudogenes participate in the regulation of variety of biological processes including cancer [105, 148, 154]. One of the earliest indications of the functional role of pseudogenes was demonstration that in mouse oocytes pseudogene-derived small interfering RNAs regulate gene expression [188, 204]. Besides fully functionally active pseudogenes, partially active pseudogenes in the process of either losing or gaining function are described [147].

The authors who study pseudogenes come to conclusion that pseudogenes serve as a source of novel functions for the evolving organisms [10, 22, 105]. A special term – “potogenes – was generated to designate pseudogenes as DNA sequences with a potentiality for becoming new genes [10, 22]. This is in accordance with the major postulate of original hypothesis of evolution by gene duplication [131], and we may consider pseudogenes with novel or evolving functions as evolutionarily novel or evolving genes.

Transcription of pseudogenes is an important indication of their functionality. The evidence of pseudogenes transcription was accumulating during the last years [10, 219]. The ENCODE and GENCODE projects provided information about transcription of 876 pseudogenes including 531 processed and 345 duplicated pseudogenes [147]. The other group of authors studied RNA-Seq transcriptome data from 248 cancer and 45 benign samples of 13 different tissue types and described the expression of 2,082 distinct pseudogenes [78]. What is important for our consideration of expression of evolutionarily novel genes in tumors, they observed 218 pseudogenes expressed only in cancer samples, of which 178 were observed in multiple cancers [78].

One of the first demonstrations that pseudogenes are activated in tumors was description of the new tumor antigen (NA88-A) generating an HLA class I-restricted CTL response against melanoma coded for by a processed pseudogene [126]. At the same time, the expression of parental gene *HPX42B* did not lead to similar CTL response. The transcription of *NA88-A* pseudogene was limited with significant expression found only in some metastatic melanomas [126].

Among other earlier works was detection of *ψPTEN* expression in central nervous system high-grade astrocytic tumors [211]. The *ψPTEN* expression was complementary to *PTEN* mutation because the majority of glioblastomas showed either *PTEN* mutation or *ψPTEN* expression. In the later study [153] the functional relationship between the mRNAs produced by the *PTEN* tumor suppressor gene and its pseudogene *PTENP1* (the other name of *ψPTEN*) was demonstrated. *PTENP1* was able to regulate cellular levels of PTEN and exerted a growth suppressive role acting as a microRNA-decoy [153].

In a comprehensive paper devoted to human processed pseudogenes Zhaolei Zhang and co-authors [217] described several pseudogene families with implication to tumors (see Table 5 in the above mentioned paper).

Other examples of pseudogenes expressed in tumors but not in normal tissues are presented in Table 2.

**Table 2:** Pseudogenes expressed in tumors

Pseudogene name	Pseudogene description	References
*HERV-K-MEL*	Expressed in melanomas, sarcomas, lymphomas, bladder and breast cancers, but not in normal tissues	[30, 169]
*CYP4Z2P*	A pseudogene of mammary-restricted cytochrome *CYP4Z1*, shows 4.8 times higher transcription level in breast cancer tissue than in normal mammary gland with almost no transcription in other tissues	[157]
*ΨCx43*	*Connexin43* (*CX43*) pseudogene was shown to be expressed in mammary cancer cell lines and to inhibit growth. *ΨCx43* acts as a posttranscriptional regulator of *CX43* in breast cancer cells	[17, 79]
*rac1*	The *rac1* processed pseudogene overexpression was detected in the human brain tumors as compared to normal brain tissues	[69]
*Oct4-pg5* *Oct4-pg1* *NANOGP8*	*Oct4* and *Nanog*, embryonic stem cell-specific genes coding for transcription factors, have multiple retropseudogenes, expressed in many cancer tissues but not in normal tissues.	[71, 73, 137, 184, 213]
*POU5F1P1* *POU5F1B* *OCT4-pg3 OCT4-pg4*	A processed pseudogene *POU5F1P1* (*POU5F1* is another name of *Oct4*) is overexpressed in prostatic carcinoma, and *OCT4-pg1*, *OCT4-pg3* and *OCT4-pg4* were found to be expressed in glioma and breast carcinoma. *POU5F1B* (also known as *Oct4-pg1* and *POU5F1P1*) is amplified and promotes aggressive phenotype to gastric cancer.	[62, 81, 206, 218]
*CRIPTO3*	A presumed pseudogene expressed in many cancer samples	[183]
*BRAF*	Expression of *BRAF* pseudogene was described in thyroid tumors.	[220]
*CSNK2A1P*	Protein kinase *CKα* intronless gene *CSNK2A1P*, a presumed *CK2α* pseudogene, plays the oncogenic role in lung cancer. It is amplified and over-expressed in non-small cell lung cancer and leukemia cell lines and in lung cancer tissues.	[67]
*MYLKP1*	A transcribed pseudogene *MYLKP1* is strongly expressed in cancer cells and its parental gene *MYLK* is highly expressed in non-neoplastic cells.	[60]
*MO1P3*	*SUMO1P3* pseudogene-expressed lncRNA is up-regulated in gastric cancer tissues and may be a potential biomarker in the diagnosis of gastric cancer	[122]
*HMGA1P6 HMGA1P7*	The overexpression of *HMGA1* pseudogenes *HMGA1P6* and *HMGA1P7* is induced in human pituitary tumors and anaplastic thyroid carcinomas.	[47, 48]
*CGB1* *CGB2*	*CGB1* and *CGB2* are transcribed in ovarian cancer tissues and in epithelial cancer cell lines	[25, 98, 187]
*ψPPM1K*	Transcribed pseudogene *ψPPM1K* exerts tumor-suppressor activity in hepatocellular carcinoma by generation of siRNA	[31]

As we can see from the data presented in this part of the paper, the expression of pseudogenes in tumors is widespread. Thus the evolution of pseudogene towards functional novel gene may involve its expression in tumors as a part of the whole process (see [91] for more discussion of the role of gene expression in the origin of novel genes).

#### Endoretroviral sequences and other retrotransposons are expressed in tumors

Transposable elements are classified in two groups, Class I and Class II. Class I mobile elements use RNA intermediate and reverse transcriptase activity for transposition, while Class II elements use a DNA intermediate and a ‘cut and paste’ mechanism. Class I elements include long terminal repeat (LTR) retrotransposons, also called ‘endogenous retroviruses’ (ERVs), and non-long terminal repeat (non-LTR) retrotransposons (LINEs and SINEs) [155]. Human transposable elements comprise about 40 % of the human genome: HERVs, 4.64 %; MaLR, 3.65 %; LINEs, 20.42 %; and SINEs, 13.14 % [100]. That is why mobile elements were called the “drivers of genome evolution” [83]. The role of transposons in gene origin was recently reviewed in [91].

Endogenous retroviruses (ERVs) have been shown to have originated as the result of repeated germ cell retroviral infection of their ancestral hosts [13, 19, 63, 118, 205]. The genes of ERVs were evolutionarily new for their ancestral hosts. Together with other retrotransposons, ERVs participated in the origin of genes with the novel functions to their hosts (reviewed in [91]). There are 203,000 copies of human ERVs (HERVs) in the human genome [100]. Different authors define different numbers of HERV families, from 26 [53] to about 50 [114, 121] or even 350 families [136].

Human endogenous retrovirus sequences are expressed in tumors [5, 111, 167]. Expression of different HERVs was described in different human tumors: HERV-K family – in teratocarcinoma [20], seminomas [167], in breast cancer [200], in urothelial and renal cell carcinomas [49], in melanoma, germ cell tumors, gonadoblastoma, ovarian clear cell carcinoma, ovarian epithelial tumors, prostate cancer, lymphoma, hematological neoplasms, sarcoma, bladder and colon cancer [30, 65, 82]; HERV-E – in prostate carcinoma [201]; HERV-H – in leukemia cell lines [107] and in cancers of small intestine, bone marrow, bladder, cervix, stomach, colon and prostate [178].

Recent reviews confirm the upregulation of HERVs in tumors [80, 113, 127, 158, 161], which is connected with general trend of HERVs demethylation in tumors [127, 158], and similar data continue to accumulate [26, 181, 208]. ERVs of mice also demonstrate hypomethylation and transcriptional upregulation in mice tumors [66, 112, 158].

Endogenous retroviruses may serve as targets for antitumor immunity. For example, *HERV-K-MEL*, a *HERV-K* pseudogene expressed in most melanomas and in many other types of tumors, encodes the antigenic peptide that is targeted by CTLs in melanoma patients [30, 169]. HERV-E was found to be selectively expressed in clear kidney cell cancer but not in normal tissues. This tumor-specific expression is connected with inactivation of the von Hippel-Lindau tumor suppressor and hypomethylation. Antigens encoded by HERV-E are immunogenic and stimulate cytotoxic T-cells that kill cancer cells. HERV proteins that act as tumor-associated antigens have also been detected in other types of tumors [37].

Especially interesting for my consideration is HERV-K family because it contains the most recently active members that entered the ancestral human genome after the divergence of humans and chimps and may be considered as evolutionarily novel for humans [12, 13, 185]. Many HERV-K proviruses are unique to humans [12]. HERV-K continued to replicate in human lineage until at least 250,000 years ago [114, 117], and might still expand [113]. HERV-K is also most widely expressed in different tumors (see above). In HERV-K and in other younger families such as HERV-H and HERV-W the most pronounced DNA demethylation was reported [49, 158]. Not only mRNA, but also HERV-K antibodies are already elevated in the blood at the early stage of breast cancer [202, 203].

RNA transcripts from various HERV LTRs have been described in various types of human tumors and cell lines. For example, elevated HERV-K 5′LTR mRNA was detected in prostate cancer tissues (reviewed in [207]).

Other primate-specific retrotransposons such as SVA, LINE-1P, AluY, and MaLR families are also known for the loss of DNA methylation in tumors. The younger retroelements are highly methylated in healthy tissues, while in many tumors these young elements suffer the most dramatic loss of methylation [49, 130, 186]. L1 and Alu sequences are silenced in normal human cells and activated in tumors [14, 155, 171]. Full length L1 RNA in cancer cell lines and expression of ORF1p in tumors have been shown (reviewed in [130]). The majority of the retrotransposition events seem to be harmless “passenger” mutations [191].

There are *in silico* data supporting the increased transcription of retrotransposons in transformed human cells [41]. Although originally it was thought that HERVs are transcriptionally silent in most normal tissues, *in silico* [57, 84, 166, 178] and PCR and microarray [6, 50, 140, 174, 179] data suggest that HERV-derived RNAs are more widely expressed in normal tissues than originally anticipated. HERV-K is transcribed during normal human embryogenesis [56]. Syncytin, the envelope gene of human defective endogenous retrovirus HERV-W, is expressed in multinucleated placental syncytiotrophoblasts and may mediate placental cytotrophoblast fusion [18, 123, 198].

#### Genes originated by exon shuffling are expressed in tumors and may lead to oncogenic transformation

The principle of gene origin by exon shuffling is the following: new genes are created by recombining previously existing exons that leads to the origin of mosaic genes and proteins [54, 75, 110, 141–143]. The exon shuffling is important mode of the origin of new genes: at least 19% of the exons in data base were involved in exon shuffling [109]. The correlation between exon-intron organization of the gene and the domain organization of the corresponding protein is most evident in the case of young vertebrate genes, e.g. genes coding for proteases of blood coagulation, fibrinolytic and complement cascades, etc. That is why the first evidence for exon shuffling came from studies on proteases of blood coagulation and fibrinolysis [143].

The mechanisms of exon shuffling include illegitimate recombination [192, 193], retroposition [125], segmental duplication [45] and L1 retrotransposon-mediated 3′ transduction [125].

Modular domain rearrangements can lead to cancer. The fusion of the self-oligomerizing SAM domain from the gene *TEL* to the catalytic domain of the nonreceptor tyrosine kinase Abl in some human leukemias results in constitutively clustered chimeric protein, persistent activation of tyrosine kinase and oncogenic transformation. Tyrosine kinases other than Abl are also activated in fusion proteins by oligomerization of SAM domain of TEL [106]. Activation of Abl tyrosine kinase seen in patients with chronic myelogenous leukemia is caused by translocation of the tip of chromosome 9 encoding Abl to chromosome 22 encoding BCR and formation of fusion protein. Oligomerization of coiled-coil domains from BCR leads to constitutive activation of Abl [106].

The *Tre2(USP6)* oncogene is a hominoid-specific gene. It originated by the fusion of two genes, *USP32* (*NY-REN-60*) and *TBC1D3. USP32* is an ancient gene and highly conserved. *TBC1D3* is young and originated by recent segmental duplication in primates. *Tre2* is young for humans as far as it originated 21–33 million years ago after *TBC1D3* segmental duplication in primates [144].

Atypical splicing in combination with retrotransposition may also lead to exon shuffling. Moreover atypical splicing of existing genes may be the most prevalent mechanism of novel protein creation. Atypical splicing includes alternative splicing within the single-gene transcripts and intergenic splicing of transcripts from tandemly located genes. Transcription-induced chimeras may evolve into gene fusions, and alternative splicing may evolve to gene fission (reviewed in [8]). For instance, the chimeric *PIPSL* gene was formed by L1-mediated retrotransposition of a readthrough, intergenically spliced transcript in hominoids [9]. This phenomenon was called transcription-mediated gene fusion. Many examples of intergenic splicing have been described in the human genome. The authors suggest that it is a novel mechanism of gene origin, where transcription-induced chimerism followed by retroposition may result in new gene [2]. At least 4 %–5 % of the tandem gene pairs in the human genome can be transcribed into a single RNA coding for chimeric protein [139].

Alternative splicing often participates in exonization process. When the new exon is alternatively spliced and expressed at low levels, splice variants with and without new exon are represented, and the pre-existing function is not destroyed. This opens the way to the origin of new gene with a new function and/or new functional module due to novel exon [54, 128, 177, 199]. The comparison of human, mouse and rat genomes indicates that alternative splicing is associated with an increased frequency of exon creation and/or loss [124].

Transposed element exonization may be a source of new constitutively spliced exons. Alu-containing exons are alternatively spliced. Comparative analysis of transposed element insertion within human and mouse genomes reveals Alu’s unique role in shaping the human transcriptome [172, 176].

The alternative splicing is widespread in cancer. The splice changes in cancer are global. Up to half of all alternative splicing events may be changed in tumors. Some splice isoforms are upregulated in all studied cancers, the others are characteristic to certain types of tumors. Affected proteins include transcription factors, cell signal transducers, transmembrane proteins, secreted extracellular proteins, proteins involved in metabolism, angiogenesis, apoptosis, cell motility and invasion, oncoproteins and tumor suppressor proteins. Genes with alternative transcripts associated with various cancers include *CD44, p53, p73, PTEN, APC, BCL-X, VEGF4, mdm2, BRCA1, TACC1, TERT, KLF6, SURVIVIN, ASIP, NF1, Caspase 8, CDH17, Ron, BARD1, AR, FGFR2, RUNX1, HOXA9, WT1, BIM, TF,* HERV-K *env* (*np9*), *HNRPK* and many others. Many of these genes have multiple splicing patterns, e.g. *mdm2* gene locus produces over 72 *mdm2* variants. Alternative splicing in cancer-related genes may have impact on all major aspects of tumor cell biology. All hallmarks of cancer have alternatively spliced regulators. There are also many cancer-associated splice variants with unknown functions [7, 35, 42, 52, 59, 85, 101, 102, 133, 156, 160, 182, 195, 196].

Atypical splicing events do not alter the number of genes in DNA, but produce altered proteins which influence all aspects of tumor biology. In evolutionary perspective, atypical splicing combined with retrotransposition may lead to the origin of novel genes. The promising direction of research would be to study what proportion of spicing events involved in cancer have already generated (through retroposition) novel genes in the germ plasm.

#### Genes originated *de novo* are specifically expressed in tumors

“Senseless” DNA sequences may acquire new functions in the organism and become new genes. New functions may be connected not only with protein-coding genes, but also with various functional non-coding RNAs. This mechanism of novel genes origin is called *de novo* origin.

New promoter elements such as GC-islands, TATA-boxes, LINE1 promoters or retroviral LTRs may arise as a result of mutational process, gene rearrangements, retrotransposition or viral infection. Such events can lead to expression of “senseless” DNA sequences that subsequently may accumulate mutations that alter their protein-coding capacity. The senseless DNA sequences acquire new functions. Noncoding RNAs may eventually acquire ORFs and become protein-coding mRNAs. These could be mechanisms of *de novo* gene origin. Exonization by alternative splicing may be the mechanism of *de novo* exon origin (see discussion above in Genes originated by exon shuffling are expressed in tumors and may lead to oncogenic transformation).

Three novel human protein-coding genes have been shown to originate from noncoding DNA since the divergence with chimp. These genes have no protein-coding homologs in any other genome. Few human-specific mutations altered protein-coding capacity by destroying “disablers” in the ancestral sequences. The existence of protein-coding genes is supported by expression and proteomic data [86]. One of those genes – *CLLU1* – has been shown earlier to be specifically expressed in chronic lymphocytic leukemia (CLL) [23]. The CLL expression specificity of *CLLU1* was later confirmed in several studies [24, 74, 134, 159]. It was also shown that *CLLU1* is expressed in other tumors (tumors of lung, stomach, prostate and spleen), but in no normal tissue [[97], in press]. We may conclude that *CLLU1* belongs to *TSEEN* genes.

*PBOV1*, a gene of the recent *de novo* origin specific to humans, has highly tumor-specific expression profile [165] (see discussion above in PBOV1, de novo originated human gene with tumor-specific expression).

*PBOV1* expression levels positively correlate with relapse-free survival in breast cancer patients and with overall longitude of survival in glioma patients [165]. On the contrary, *CLLU1* is highly expressed in poor-prognostic patients [23, 24, 74, 134, 159].

#### Positive selection of human tumor-related genes in primate lineage

Positive Darwinian selection participates in the evolution of the novel genes. Comparison of the rate of amino acid replacement substitution with the rate of synonymous substitution, population genetic analyses of polymorphisms and the findings of convergent evolution support the adaptive evolution of the novel genes. There are many examples of rapidly evolving novel genes and gene families supported by positive selection. In humans, strong positive selection and accelerated evolution was documented for lactase gene and for many other genes with different molecular functions, e.g. transcription factors, genes involved in nuclear transport, DNA metabolism/cell cycle, protein metabolism, pigmentation pathways, dystrophin protein complex, heat shock proteins; various types of genes related to sensory perception, immune response, reproduction, morphology, host-pathogen interactions, and neuronal functions. Examples of positively selected gene families are also numerous, including those in African great apes and hominids. Several gene families have expanded or contracted rapidly in primates, including brain-related families in humans. Many of such families show evidence for positive selection. The proportion of positively selected genes is significantly higher in younger genes in humans, i.e. positive selection may play a role in faster evolution of younger genes. Many examples of rapid evolution and positive selection of new genes described in the literature points out that this phenomenon is widespread. It supports involvement of novel genes and gene families in adaptation and speciation and in evolution and enhancement of new functions (reviewed in [91]).

For our consideration, it is important that positive selection in primate lineage was described for many human tumor-related genes [39, 40, 43, 129, 145, 180].

*SPANX*, *GAGE*, *PRAME* and *CTAGE* families of cancer/testis antigen genes, with unknown functions yet, undergo positive selection in primate evolution [43, 55, 87, 108, 214]. Comparison of human/chimp orthologues of CT-X genes has shown that they diverge faster and undergo stronger positive selection than those on the autosomes [180].

Adaptive evolution of the tumor suppressor BRCA1 in humans and chimps was demonstrated [68]. Most of the internal BRCA1 sequence is variable between primates and evolved under positive selection [145].

Angiogenin (ANG) is the tumor-growth promoter due to its ability to stimulate the formation of new blood vessels. Its expression is elevated in variety of tumors. The study among several primate species showed that *ANG* gene has a significantly higher rate of nucleotide substitution at nonsynonymous site than at synonymous sites, an indication of positive selection [212].

Comparison of 7645 chimp gene sequences with their human and mouse orthologs showed accelerated evolution in functions related to oncogenesis [39]. A search for positively selected genes in the genomes of humans and chimps showed the evidence for positive selection in many genes involved in tumor suppression, apoptosis and cell cycle control [129].

More examples of positively selected tumor-related genes are reviewed in [40].

Positive selection of many human tumor-related genes in the evolution of primates confirms the prediction of evolution by tumor neofunctionalization hypothesis concerning expression of evolutionarily new genes in tumors and selection for their new organismal functions. If an evolutionarily new gene is expressed in tumors, or a sequence that is expressed in tumors acquires a function beneficial to the organism and becomes an evolutionarily new gene, selection of organisms for the enhancement of the new function should take place, as predicted by the hypothesis. This is exactly what was found in papers discussed above: the positive selection of genes and proteins in different primate groups, not the somatic evolution of tumor cells. More discussion of positive selection in relation to the possible evolutionary role of tumors may be found in [91].

The paradox of the positive selection of many tumor-associated genes is difficult to explain otherwise than by the postulation that tumors play a positive evolutionary role. The other attempt to explain positive selection of tumor-related genes is based on the concept of genomic conflict and antagonistic coevolution [40, 129].

Some evolutionarily novel genes are cellular oncogenes. The *Tre2(USP6)* oncogene is a hominoid-specific gene [144] (see discussion above in part 2.3). Evolutionarily novel genes *CT45A1*, *TBC1D3* and *NCYM* may act like oncogenes (reviewed in [215]). Y. Zhang and M. Long suggest that these genes may also assume other biological functions, and attract the selection, pleiotropy and compensation hypothesis of M. Pavlicev and G.P. Wagner [146] to explain the paradox related to their oncogene role.

## Conclusion

### The phenomenon of tumor specifically expressed, evolutionarily novel genes (*TSEEN* genes)

This review discusses the data obtained in my lab and the data described in the literature. My group looked for genes with dual specificity, i.e. evolutionarily novel and tumor specifically expressed. We studied single genes, the complex class of CT genes with many gene families, and two newly described gene classes obtained by global subtraction of normal cDNA sequences from tumor cDNA sequences. Using different approaches, we have been able to describe many genes with tumor specific or tumor predominant expression which are also evolutionarily novel or young.

We have also described tumor-specifically expressed, evolutionarily new sequences which look like proto-genes, i.e. gene precursors which have not yet acquired functions and evolve neutrally. Expression of proto-genes, novel and young genes in tumors may represent different stages of the origin of a new genes and novel organismal functions (which are not related to tumor progression) in multicellular organisms.

The analysis of published information about evolutionarily novel genes and/or sequences originated through different molecular mechanisms (by gene duplication, from endogenous viruses and retrotransposons, by exon shuffling or *de novo*) reveals that evolutionarily novel genes/sequences tend to be expressed predominantly in tumors, independent of the mechanism of origin. Sometimes the expression of evolutionarily novel genes in tumors is highly specific. Moreover, positive selection of many human tumor-related genes in primate lineage suggests their involvement in the origin of new functions beneficial to organisms.

I suggested considering the expression of evolutionarily young or novel genes in tumors as a new biological phenomenon, a phenomenon of *TSEEN* (tumor specifically expressed, evolutionarily novel) genes [91]. This phenomenon is similar to phenomenon of carcinoembryonic antigens in that it represents a phenomenon of dual specificity, i.e. evolutionary and tumor specificities.

Some *TSEEN* genes are oncogenes, the others acquired functions beneficial to organism, but many *TSEEN* genes have no known functions. The lack of know functions is usually associated with the youngest *TSEEN* genes. We may infer that they are in the process of acquisition of function in the organism as suggested by positive selection of many of them in primate lineage.

*TSEEN* genes may thus represent a new interesting link between different but connected processes of gene origin, genome evolution, tumorigenesis and progressive evolution.

## References

[1] Akers SN, Odunsi K, Karpf AR (2010). Regulation of cancer germline antigen gene expression: implications for cancer immunotherapy. Future Oncol.

[2] Akiva P, Toporik A, Edelheit S, Peretz Y, Diber A, Shemesh R, Novik A, Sorek R (2006). Transcription-mediated gene fusion in the human genome. Genome Res.

[3] Altschul S, Gish W, Miller W, Myers E, Lipman D (1990). Basic local alignment search tool. J Mol Biol.

[4] An G, Ng AY, Meka CSR, Luo G, Bright SP (2000). Cloning and characterization UROC28, a novel gene overexpressed in prostate, breast and bladder cancers. Cancer Res.

[5] Anderson A, Svensson A, Rolny C (1998). Expression of human endogenous retrovirus ERV3 (HERV-R) mRNA in normal and neoplastic tissues. Int J Oncol.

[6] Andersson A-C, Yun Z, Sperber GO, Larsson E, Blomberg J (2005). ERV3 and related sequences in humans: structure and RNA expression. J Virol.

[7] Armbruester V, Sauter M, Krautkraemer E (2002). A novel gene from the human endogenous retrovirus K expressed in transformed cells. Clin Cancer Res.

[8] Babushok DV, Ostertag EM, Kazazian HH Jr. Current topics in genome evolution: Molecular mechanisms of new gene formation. Cell Mol Life Sci. 2006. doi: 10.1007/s00018-006-6453-4 10.1007/s00018-006-6453-4 PMC11138463 17192808

[9] Babushok DV, Ohshima K, Ostertag EM, Chen X, Wang Y, Mandal PK, Okada N, Abrams CS, Kazazian HH (2007). A novel testis ubiquitin-binding protein gene arose by exon shuffling in hominoids. Genome Res.

[10] Balakirev ES, Ayala FJ (2003). Pseudogenes: are they “junk” or functional DNA?. Annu Rev Genet.

[11] Baranova AV, Lobashev AV, Ivanov DV, Krukovskaya LL, Yankovsky NK, Kozlov AP (2001). In silico screening for tumor-specific expressed sequences in human genome. FEBS Lett.

[12] Barbulescu M, Turner G, Seaman MI, Deinard AS, Kidd KK, Lenz J (1999). Many human endogenous retrovirus K (HERV-K) proviruses are unique to humans. Curr Biol.

[13] Belshaw R, Pereira V, Katzourakis A, Talbot G, Paces J, Burt A (2004). Long-term reinfection of the human genome by endogenous retroviruses. Proc Natl Acad Sci U S A.

[14] Berdasco M, Esteller M (2010). Aberrant epigenetic landscape in cancer: How cellular identity goes awry. Dev Cell.

[15] Betran E, Long M (2003). *Dntf-2r*, a young Drosophila retroposed gene with specific male expression under positive Darwinian selection. Genetics.

[16] Betran E, Thornton K, Long M (2002). Retroposed new genes out of the X in Drosophila. Genome Res.

[17] Bier A, Oviedo-Landaverde I, Zhao J, Mamane Y, Kandouz M, Batist G. *Connexin43* pseudogene in breast cancer cells offers a novel therapeutic target. Mol. Cancer Ther. 2009;8(4). doi: 10.1158/1535-7163.MCT-08-0930 10.1158/1535-7163.MCT-08-0930 19372551

[18] Blond J-L, Beseme F, Duret L, Bouton O, Bedin F, Perron H, Mandrand B, Mallet F (1999). Molecular characterization and placental expression of HERV-W, a new human endogenous retrovirus family. J Virol.

[19] Boeke JD, Stoye JP, Coffin JM, Hughes SH, Varmus HE (1997). Retrotransposons, endogenous retroviruses, and the evolution of retroelements. Retroviruses.

[20] Boller K, Konig H, Sauter M, Mueller-Lantzsch N, Lower R, Lower J, Kurth R (1993). Evidence that HERV-K is the endogenous retrovirus sequence that codes for the human teratocarcinoma-derived retrovirus HTDV. Virology.

[21] Brosius J (1999). RNAs from all categories generate retrosequences that may be exapted as novel genes or regulatory elements. Gene.

[22] Brosius J, Gould SJ (1992). On “genomenclature”: A comprehensive (and respectful) taxonomy for pseudogenes and other “junk DNA”. Proc Natl Acad Sci U S A.

[23] Buhl AM, Jurlander J, Jorgensen FS (2006). Identification of a gene on chromosome 12q22 uniquely overexpressed in chronic lymphocytic leukemia. Blood.

[24] Buhl AM, Jurlander J, Geisler CH (2006). CLLU1 expression levels predict time to initiation of therapy and overal survival in chronic lymphocytic leukemia. Eur J Haematol.

[25] Burczynska BB, Kobrouly L, Butler SA, Naase M, Iles RK (2014). Novel insights into the expression of *CBG1 & 2* genes by epithelial cancer cell lines secreting ectopic free hCGβ. Anticancer Res.

[26] Buslei R, Strissel PL, Henke C, Schey R, Lang N, Ruebner M, Stolt CC, Fabry B, Buchfelder M, Strick R (2015). Activation and regulation of endogenous retroviral genes in the human pituitary gland and related endocrine tumors. Neuropathol Appl Neurobiol.

[27] Caballero OL, Chen Y-T (2009). Cancer/testis (CT) antigens: potential targets for immunotherapy. Cancer Sci.

[28] Capra JA, Williams AG, Pollard KS (2012). ProteinHistorian: Tools for the Comparative Analysis of Eukaryote Protein Origin. PLoS Comput Biol.

[29] Carvunis A-R, Rolland T, Wapinski I, Calderwood MA (2012). Proto-genes and *de novo* gene birth. Nature.

[30] Cegolon L, Salata C, Weiderpass E, Vineis P, Palu G, Mastrangelo G (2013). Human endogenous retroviruses and cancer prevention: evidence and prospects. BMC Cancer.

[31] Chan W-L, You C-Y, Yang W-K, Hung S-Y (2013). Transcribed pseudogene *ψPPM1K* generates endogenous siRNA to suppress oncogenic cell growth in hepatocellular carcinoma. Nucl Acids Res.

[32] Chang T-C, Yang Y, Yasue H, Bharti AK, Retzel EF, Liu W-S (2011). The expansion of the *PRAME* gene family in Eutheria. PLoS One.

[33] Chen Y-T, Iseli C, Venditti CA, Old LJ, Simpson AJG, Jongeneel CV (2006). Identification of a new cancer/testis gene family, *CT47*, among expressed multicopy genes on the human X chromosome. Genes Chrom Cancer.

[34] Chen S, Krinsky BH, Long M (2013). New genes as drivers of phenotypic evolution. Nature Rev.

[35] Chen J, Weiss WA (2014). Alternative splicing in cancer: implications for biology and therapy. Oncogene 2015.

[36] Cheng Y-H, Wong EWP, Cheng CY (2011). Cancer/testis (CT) antigens, carcinogenesis and spermatogenesis. Spermatogenesis.

[37] Cherkasova E, Weisman Q, Childs RW (2013). Endogenous retroviruses as targets for antitumor immunity in renal cancer and other tumors. Front Oncol.

[38] Clamp M, Fry B, Kamal M, Xie X, Cuff J, Lin MF, Kellis M, Lindblad-Toh K, Lander ES (2007). Distinguishing protein-coding and noncoding genes in the human genome. Proc Natl Acad Sci U S A.

[39] Clark AG, Glanowski S, Nielsen R, Thomas PD, Kejariwal A, Todd MA (2003). Inferring nonneutral evolution from human-chimp-mouse orthologous gene trios. Science.

[40] Crespi BJ, Summers K (2006). Positive selection in the evolution of cancer. Biol Rev.

[41] Criscione SW, Zhang Y, Thompson W, Sedivy JM, Neretti N (2014). Transcriptional landscape of repetitive elements in normal and cancer cells. BMC Genomics.

[42] David CJ, Manley JL (2010). Alternative pre-mRNA splicing regulation in cancer: pathways and programs unhinged. Genes Dev.

[43] Demuth JP, Hahn MW (2009). The life and death of gene families. BioEssays.

[44] Dobrynin P, Matyunina E, Malov SV, Kozlov AP (2013). The novelty of human cancer/testis antigen encoding genes in evolution. Int J Genomics.

[45] Eichler EE (2001). Recent duplication, domain accretion and the dynamic mutation of the human genome. Trends Genet.

[46] Emerson JJ, Kaessmann H, Betran E, Long M (2004). Extensive gene traffic on the mammalian X chromosome. Science.

[47] Esposito F, De Martino M, Forzati F, Fusco A (2014). *HMGA1*-pseudogene overexpression contributes to cancer progression. Cell Cycle.

[48] Esposito F, De Martino M, D’Angelo D, Mussnich P (2015). *HMGA1*-pseudogene expression is induced in human pituitary tumors. Cell Cycle.

[49] Florl AR, Lower R, Schmitz-Drager BJ, Schulz WA (1999). DNA methylation and expression of LINE-1 and HERV-K provirus sequences in urothelial and renal cell carcinomas. British J Cancer.

[50] Frank O, Giehl M, Zheng C, Hehlmann R, Leib-Mosch C, Seifarth W (2005). Human endogenous retrovirus expression profiles in samples from brains of patients with schizophrenia and bipolar disorders. J Virol.

[51] Galachyants Y, Kozlov AP (2009). CDD as a tool for discovery of specifically-expressed transcripts. Russ J AIDS, Cancer Public Health.

[52] Ghigna C, Valacca C, Biamonti G (2008). Alternative splicing and tumor progression. Curr Genomics.

[53] Gifford R, Tristem M (2003). The evolution, distribution and diversity of endogenous retroviruses. Virus Genes.

[54] Gilbert W (1978). Why genes in pieces?. Nature.

[55] Gjerstorff MF, Ditzel HJ (2008). An overview of the GAGE cancer/testis antigen family with the inclusion of newly identified members. Tissue Antigens.

[56] Grow EJ, Flynn RA, Chavez SL (2015). Intrinsic retroviral reactivation in human preimplantation embryos and pluripotent cells. Nature.

[57] Haase K, Mosch A, Frishman D (2015). Differential expression analysis of human enogenous viruses based on RNCODE RNA-seq data. BMC Med Genet.

[58] Hahn MW (2009). Distingwishing among evolutionary models for the maintenance of gene duplicates. J Hered.

[59] Hahn CN, Venugopal P, Scott HS, Hiwase DK (2014). Splice factor mutations and alternative splicing as drivers of hematopoietic malignancy. Immunol Rev.

[60] Han YJ, Ma SF, Yourek G, Park Y-D, Garcia GN (2011). A transcribed pseudogene of *MYLK* promotes cell proliferation. FASEB J.

[61] Harrow J, Frankish A, Gonzalez JM (2012). GENCODE: the reference human genome annotation for The ENCODE Project. Genome Res.

[62] Hayashi H, Arao T, Togashi Y (2013). The *OCT4* pseudogene *POU5F1B* is amplified and promotes an aggressive phenotype in gastric cancer. Oncogene.

[63] Hayward A, Cornwallis CK, Jern P (2015). Pan-vertebrate comparative genomics unmasks retrovirus macroevolution. Proc Natl Acad Sci U S A.

[64] Hofmann O, Caballero OL, Stevenson BJ, Chen Y-T, Cohen T, Chua R, Maher CA, Panji S, Schaefer U, Kruger A, Lehvaslaiho M, Carninci P, Hayashizaki Y, Jongeneel CV, Simpson AJG, Old LJ, Hide W (2008). Genome-wide analysis of cancer-testis gene expression. Proc Natl Acad Sci U S A.

[65] Hohn O, Hanke K, Bannert N (2013). HERV-K(HML-2), the best preserved family of HERVs: endogenization, expression, and implications in health and disease. Frontiers in Oncology.

[66] Howard G, Eiges R, Gaudet F, Jaenish R, Eden A (2008). Activation and transposition of endogenous retroviral elements in hypomethylation induced tumors in mice. Oncogene.

[67] Hung M-S, Lin Y-C, Mao J-H, Kim I-J (2010). Functional polymorphism of the CK2α intronless gene plays oncogenic roles in lung cancer. PLoS One.

[68] Huttley G.A., Easteal S., Southey M.C., Tesoriero A., Giles G.G., McCredie M.R.E., Hopper J.L., Venter D.J., and the Australian Breast Cancer Family Study (2000). Adaptive evolution of the tumor suppressor *BRCA1* in in humans and chimpanzees. Nature Genet.

[69] Hwang SL, Chang JH, Cheng CY, Howng SL, Sy WD, Lieu AS, Lin CL, Lee KS, Hong YR (2005). The expression of *rac1* pseudogene in human tissues and in human brain. Eur Surg Res.

[70] Innan H, Kondrashov F (2010). The evolution of gene duplications: classifuing and distinguishing between models. Nature Rev Genet.

[71] Ishiguro T, Sato A, Ohata H (2012). Differential expression of *nanog1* and *nanogp8* in colon cancer cells. Biochem Biophys Res Commun.

[72] Jacq C, Miller JR, Brownlee GG (1977). A pseudogene structure in 5S DNA of *Xenopus laevis*. Cell.

[73] Jeter CR, Badeaux M, Choy G (2009). Functional evidence that the self-renewal gene *NANOG* regulates human tumor development. Stem Cells.

[74] Josefson P, Geisler CH, Leffers H (2007). CCLU1 expression analysis adds prognostic information to risk prediction in chronic lymphocytic leukemia. Blood.

[75] Kaessmann H, Zollner S, Nekrutenko A, Li WH (2002). Signatures of domain shuffling in the human genome. Genome Res.

[76] Kaessmann H, Vinckenbosch N, Long M (2009). RNA-based gene duplication: mechanistic and evolutionary insights. Nature Rev.

[77] Kaessmann H (2010). Origins, evolution, and phenotypic impact of new genes. Genome Res.

[78] Kalyana-Sundaram S, Kumar-Sinha C, Shankar S (2012). Expressed pseudogenes in the transcriptional landscape of human cancers. Cell.

[79] Kandouz M, Bier A, Carystinos GD, Alaoui-Jamali MA, Batist G (2004). Connexin43 pseudogene is expressed in tumor cells and inhibits growth. Oncogene.

[80] Kassiotis G (2014). Endogenous retroviruses and the development of cancer. J Immunol.

[81] Kastler S, Honold L, Luedeke M (2010). *POU5F1P1*, a putative cancer susceptibility gene, is overexpressed in prostatic carcinoma. Prostate.

[82] Katoh I, Kurata S (2013). Association of endogenous retroviruses and long terminal repeats with human disorders. Frontiers in Oncology.

[83] Kazazian HH (2004). Mobile elements: drivers of genome evolution. Science.

[84] Kim TH, Jeon YJ, Yi JM, Kim DS, Huh JW, Hur CG, Kim HS (2004). The distribution and expression of HERV families in the human genome. Mol Cells.

[85] Kim YJ, Kim HS (2012). Alternative splicing and its impact as a cancer diagnostic marker. Genomics and Informatics.

[86] Knowles DG, McLysaght A (2009). Recent de novo origin of human protein-coding genes. Genome Res.

[87] Kouprina N, Mullokandov M, Rogozin IB, Collins NK, Solomon G, Otstot J, Risinger JI, Koonin EV, Barrett JC, Lariononv V (2004). The *SPANX* gene family of cancer/testis-specific antigens: Rapid evolution and amplification in African great apes and hominids. Proc Natl Acad Sci U S A.

[88] Kozlov AP (1996). Gene competition and the possible evolutionary role of tumors. Med Hypotheses.

[89] Kozlov AP (2008). Tumors and evolution. Vopr Onkol.

[90] Kozlov AP (2010). The possible evolutionary role of tumors in the origin of new cell types. Med Hypotheses.

[91] Kozlov AP (2014). Evolution by Tumor Neofunctionalization.

[92] Kozlov AP, Galachyants YP, Dukhovlinov IV, Samusik NA, Baranova AV, Polev DE, Krukovskaya LL (2006). Evolutionarily new sequences expressed in tumors. Infect Agent Cancer.

[93] Kozlov A, Krukovskaya L, Baranova A, Tyezelova T, Polev D (2003). Transcriptional activation of evolutionary new genes in human tumors. Russ J HIV/AIDS and Related Problems.

[94] Krukovskaya LL, Baranova A, Tyezelova T, Polev D, Kozlov AP (2005). Experimental study of human expressed sequences newly identified in silico as tumor specific. Tumor Biol.

[95] Krukovskaya LL, Nosova Yu K, Polev DK, Baranova AV, Galachyantz Yu P, Samusik NA, Kozlov AP (2007). Expression of nine tumor-associated nucleotide sequences in human normal and tumor tissues. Russ J AIDS, Cancer and Public Health.

[96] Krukovskaya LL, Samusik ND, Shilov ES, Polev DE, Kozlov AP (2010). Tumor-specific expression of PBOV1, a new gene in evolution. Vopr Onkol.

[97] Krukovskaya LL, Polev DE, Kurbatova TV, Karnauhova Yu K, Kozlov AP. The studies of tumor specificity of expression of some evolutionarily novel genes. Vopr Onkol. 2016;62(No.3), in press 30463107

[98] Kubiczak M, Walkowiak GP, Nowak-Markwitz E, Jankowska A (2013). Human chorionic gonadotropin beta subunit genes *CGB1* and *CGB2* are transcriptionally active in ovarian cancer. Int J Mol Sci.

[99] Lahn BT, Page DC (1999). Four evolutionary strata on the human X chromosome. Science.

[100] Lander ES, Linton LM, Birren B, Nusbaum C, Zody MC, Baldwin J (2001). Initial sequencing and analysis of the human genome. Nature.

[101] Leopoldino AM, Carregaro F, Silva CHTP (2007). Sequence and transcriptional study of *HNRPK* pseudogenes, and expression and molecular modeling analysis of hnRNP K isoforms. Genome.

[102] Leppert U, Eisenreich A (2015). (2014) The role of tissue factor isoforms in cancer biology. Int JCancer.

[103] Levine MT, Jones CD, Kern AD, Lindfors HA, Begun DJ (2006). Novel genes derived from noncoding DNA in Drosophila melanogaster are frequently X-linked and exhibit testis-biased expression. Proc Natl Acad Sci U S A.

[104] Li WH (1997). Molecular Evolution.

[105] Li W, Yang W, Wang XJ (2013). Pseudogenes: Pseudo or real functional elements?. J Genet Genom.

[106] Lim W, Mayer B, Pawson T. Cell signaling. Principles and mechanisms. New York: Garland Science; 2015.

[107] Lindeskog M, Blomberg J (1997). Spliced human endogenous retroviral HERV-H env transcripts in T-cell leukaemia cell lines and normal leukocytes: alternative splicing pattern of HERV-H transcripts. J Gen Virol.

[108] Liu Y, Zhu Q, Zhu N (2008). Recent duplication and positive selection of the GAGE gene family. Genetica.

[109] Long M, Rosenberg C, Gilbert W (1995). Intron phase correlation and the evolution of the intron/exon structure of genes. Proc Natl Acad Sci U S A.

[110] Long M, Betran E, Thornton K, Wang W (2003). The origin of new genes: glimpses from the young and old. Nature Rev.

[111] Lower R, Lower J, Tondera-Koch C (1993). A general method for identification of transcribed retrovirus sequences (R-U5 PCR) reveals the expression of the human endogenous retrovirus loci HERV-H and HERV-K in teratocarcinoma cells. Virology.

[112] Lueders KK, Fewell JW, Morozov VE, Kuff EL (1993). Selective expression of intracisternal A-particle genes in established mouse plasmacytomas. Mol Cell Biol.

[113] Magiorkinis G, Belshaw R, Katzourakis A (2013). ‘There and back again’: revisiting the pathophysiological roles of human endogenous retroviruses in the post-genomic era. Phil Trans R Soc B.

[114] Magiorkinis G, Blanco-Melo D, Belshaw R (2015). The decline of human endogenous retroviruses: extinction and survival. Retrovirology doi.

[115] Makashov A, Kozlov AP. The human oncogenome evolution advances ahead of the evolution of human protein-coding genome and other specific gene classes. Eur J Cancer Suppl. 2015a;13(1). http://dx.doi.org/10.1016/j.ejcsup. 2015.08.062

[116] Makashov A, Kozlov AP. Different classes of human genes have different relative evolutionary novelty. CSH-ASIA/AACR joint meeting: Big data, computation, and systems biology in cancer. 2015b

[117] Marchi E, Kanapin A, Magiorkinis G, Belshaw R (2014). Infixed endogenous retroviral insertions in the human population. J Virol.

[118] Mariani-Costantini R, Horn TM, Callahan R (1989). Ancestry of human endogenous retrovirus family. J Virol.

[119] Marques AC, Dupanloup I, Vinckenbosch N, Reymond A, Kaessmann H (2005). Emergence of young human genes after a burst of retroposition in primates. PLoS Biol.

[120] Marques-Bonet T, Girirajan S, Eichler EE (2009). The origins and impact of primate segmental duplications. Trends Genet.

[121] Mayer J, Blomberg J, Seal RL (2011). A revised nomenclature for transcribed human endogenous retroviral loci. Mob DNA.

[122] Mei D, Song H, Wang K, Lou Y, Sun W, Liu Z, Ding X, Guo J (2013). Up-regulation of SUMO1 pseudogene 3 (SUMO1P3) in gastric cancer and its clinical association. Med Oncol.

[123] Mi S, Lee X, Li X, Veldman GM, Finnerty H, Racie L, LaVallie E, Tang XY, Edouard P, Howes S (2000). Syncytin is a captive retroviral envelope protein involved in human placental morphogenesis. Nature.

[124] Modrek B, Lee CJ (2003). Alternative splicing in the human, mouse and rat genomes is associated with an increased frequency of exon creation and/or loss. Nuture Genet.

[125] Moran JV, DeBerardinis RJ, Kazazian HH (1999). Exon shuffling by L1 retrotransposition. Science.

[126] Moreau-Aubry A, Le Guiner S, Labarriere N, Gesnel MC, Jotereau F, Breathnach R (2000). A processed pseudogene codes for a new antigen recognized by a CD8(+) T cell clone on melanoma. J Exp Med.

[127] Mullins CS, Linnebacher M (2012). Human endogenous retroviruses and cancer: Causality and therapeutic possibilities. World J Gastroenterol.

[128] Nekrutenko A (2004). Identification of novel exons from rat-mouse comparisons. J Mol Evol.

[129] Nielsen R, Bustamante C, Clark AG, Glanowski S, Sackton TB, Hubisz MJ (2005). A scan for positively selected genes in the genomes of humans and chimpanzees. PLoS Biol.

[130] O’Donnell KA, Burns KH (2010). Mobilizing diversity: transposable element insertions in genetic variation and disease. Mob DNA.

[131] Ohno S (1970). Evolution by gene duplication.

[132] Ohno S (1999). Gene duplication and the uniqueness of vertebrate genomes circa 1970 – 1999. Cell Dev Biol.

[133] Oltean S, Bates DO (2013). Hallmarks of alternative splicing in cancer. Oncogene 2014.

[134] Oppliger LE, Rogenmoser-Dissler D, de Beer D (2012). CLLU1 expression distinguishes chronic lymphocytic leukemia from other mature B-cell neoplasms. Leuk Res.

[135] Oshima K, Hattori M, Yada T, Gojobori T, Sakaki Y, Okada N (2003). Whole-genome screening indicates a possible burst of formation of processed pseudogenes and Alu repeats by particular L1 subfamilies in ancestral primates. Genome Biol.

[136] Paces J, Pavlicek A, Zika R, Kapitonov VV, Jurka J, Paces V (2004). HERVd: the Human Endogenous RetroViruses Database: update. Nucleic Acids Res.

[137] Pain D, Chirn G-W, Strassel C, Kemp DM (2005). Multiple retropseudogenes from pluripotent cell-specific gene expression indicate a potential signature for novel gene identification. J Biol Chem.

[138] Palena C, Polev DE, Tsang KY, Fernando RI, Litzinger M, Krukovskaya LL, Baranova AV, Kozlov AP, Schlom J (2007). The human T-box mesodermal transcription factor Brachyury is a candidate target for T-cell-mediated cancer immunotherapy. Clin Cancer Res.

[139] Parra G, Reymond A, Dabbouseh N, Dermitzakis ET, Castelo R, Thomson TM, Antorakis SE, Guigo R (2006). Tandem chimerism as a means to increase protein complexity in the human genome. Genome Res.

[140] de Parseval N, Lazar V, Casella J-F, Benit L, Heidmann T (2003). Survey of human genes of retroviral origin: identification and transcriptome of the genes with coding capacity for complete envelope proteins. J Virol.

[141] Patthy L (1985). Evolution of the proteases of blood coagulation and fibrinolysis by assembly from modules. Cell.

[142] Patthy L (1996). Exon shuffling and other ways of module exchange. Matrix Biol.

[143] Patthy L (2003). Modular assembly of genes and the evolution of new functions. Genetica.

[144] Paulding CA, Ruvolo M, Haber DA (2003). The *Tre2* (*USP6*) oncogene is a hominoid-specific gene. Proc Natl Acad Sci U S A.

[145] Pavlicek A, Noskov V, Kouprina N, Barret JC, Jurka J, Larionov V (2004). Evolution of the tumor suppressor *BRCA1* locus in primates: implications for cancer predisposition. Hum Mol Genet.

[146] Pavlicev M, Wagner GP (2012). A model of developmental evolution: selection, pleiotropy and compensation. Trends Ecol Evol.

[147] Pei B, Sisu C, Frankish A (2012). The GENCODE pseudogene resource. Genome Biol.

[148] Pink RC, Wicks K, Caley DP, Punch EK, Jacobs L, Carter DRF (2011). Pseudogenes: Pseudo-functional or key regulators in health and disease?. RNA.

[149] Polev DE, Nosova JK, Krukovskaya LL, Baranova AV, Kozlov AP (2009). Expression of transcripts corresponding to cluster Hs.633957 in human healthy and tumor tissues. Mol Biol (Mosk).

[150] Polev DE, Krukovskaya LL, Kozlov AP (2011). Locus Hs.633957 expression in human gastrointestinal tract and tumors. Vopr Onkol.

[151] Polev D, Krukovskaia L, Karnaukhova J, Kozlov A. Transcribed locus Hs.633957: A new tumor-associated primate-specific gene with possible microRNA function. Proceedings of the 102nd Annual Meeting of the American Association for Cancer Research. Abstract No 3858. 2011b

[152] Polev DE, Karnaukhova JK, Krukovskaya LL, Kozlov AP (2014). *ELFN1-AS1* – a novel primate gene with possible microRNA function expressed predominantly in tumors. BioMed ResInt.

[153] Poliseno L, Salmena L, Zhang J, Carver B, Haveman WJ, Pandolfi PP (2010). A coding-independent function of gene and pseudogene mRNAs regulates tumor biology. Nature.

[154] Poliseno L (2012). Pseudogenes: Newly discovered players in human cancer. Sci Signal.

[155] Rahbari R, Habibi L, Garcia-Puche JL, Badge RM, Garcia-Perez J (2015). LINE-1 retrotransposons and their role in cancer.

[156] Rahmutulla B, Matsushita K, Nomura F (2014). Alternative splicing of DNA damage response genes and gastrointestinal cancers. World J Gastroenterol.

[157] Rieger MA, Ebner R, Bell DR (2004). Identification of a novel mammary-restricted cytochrome P450, CYP4Z1, with overexpression in breast carcinoma. Cancer Res.

[158] Romanish MT, Cohen CJ, Mager DL (2010). Potential mechanisms of endogenous retroviral-mediated genomic instability in human cancer. Semin Cancer Biol.

[159] Rosenquist R, Cortese D, Bhoi S (2013). Prognostic markers and their clinical applicability in chronic lymphocytic leukemia: where do we stand?. Leuk Lymphoma.

[160] Rosso M, Okoro DE, Bargonetti J. Splice variants of MDM2 in oncogenesis. In: Deb SP, Deb S editors. Mutant p53 and MDM2 in cancer, Subcellular Biochemistry 85. Springer Science + Business Media Dortrecht 2014; 2014. doi: 10.1007/978-94-017-9211-0_14 10.1007/978-94-017-9211-0_14 25201199

[161] Ruprecht K, Mayer J, Sauter M, Roemer K, Muller-Lantzsch N (2008). Endogenous retroviruses and cancer. Cell Mol Life Sci.

[162] Samusik NA, Galachyantz YP, Kozlov AP (2007). Comparative-genomic analysis of human tumor-related transcripts. Russ J AIDS, Cancer and Public Health.

[163] Samusik NA, Galachyants YP, Kozlov AP (2009). Analysis of evolutionary novelty of tumor-specifically expressed sequences. Ecologicheskaya Genetika.

[164] Samusik NA, Galachyants YP, Kozlov AP (2011). Analysis of evolutionary novelty of tumor-specifically expressed sequences. Russian J Genet: Applied Res.

[165] Samusik N, Krukovskaya L, Meln I, Shilov E, Kozlov AP (2013). PBOV1 is a human *de novo* gene with tumor-specific expression that is associated with a positive clinical outcome of cancer. PLoS One.

[166] Santoni FA, Guerra J, Luban J (2012). HERV-H RNA is abundant in human embryonic stem cells and a precise marker for pluripotency. Retrovirology.

[167] Sauter M, Schommer S, Kremmer E (1995). Human endogenous retrovirus K10: expression of gag protein and detection of antibodies in patients with seminomas. J Virol.

[168] Sayers EW, Barrett T, Benson DA (2012). Database resources of the National Center for Biotechnology Information. Nucl Acids Res.

[169] Schiavetti F, Thonnard J, Colau D, Boon T, Coulie PG (2002). A human endogenous retroviral sequence encoding an antigen recognized on melanoma by cytolytic lymphocytes. Cancer Res.

[170] Schlom J, Palena CM, Kozlov AP, Tsang K. Brachyury polipeptides and methods for use. United States Patent No. 8,188,214 B2. 2012

[171] Schulz WA (2006). L1 retrotransposons in human cancer. J Biomed Biotechnol.

[172] Sela N, Mersch B, Gal-Mark N, Lev-Maor G, Hotz-Wagenblatt A, Ast G. Comparative analysis of transposed elements’ insertion within human and mouse genomes reveals *Alu*’s unique role in shaping the human transcriptome. Genome Biol. 2007; 8: doi: 10.1186/gb-2007-8-6-r127 10.1186/gb-2007-8-6-r127 PMC2394776 17594509

[173] Sen K, Ghosh TC (2013). Pseudogenes and their composers: delving in the ‘debris’ of human genome. Briefings in Functional Genomics.

[174] Sibata M, Ikeda H, Katumata K, Takeuchi K, Wakisaka A, Yoshoki T (1997). Human endogenous retroviruses: expression in various organs in vivo and its regulation in vitro. Leukemia.

[175] Simpson AJ, Caballero OL, Jungbluth A, Chen Y-T, Old LJ (2005). Cancer/testis antigens, gametogenesis and cancer. Nat Rev Cancer.

[176] Sorek R, Ast G, Graur D (2002). *Alu*-containing exons are alternatively spliced. Genome Res.

[177] Sorek R (2007). The birth of new exons: Mechanisms and evolutionary consequences. RNA.

[178] Stauffer Y, Theiler G, Sperisen P, Lebedev Y, Jongeneel CV (2004). Digital expression profiles of human endogenous retroviral families in normal and cancerous tissues. Cancer Immun.

[179] Stengel A, Roos C, Hunsmann G, Seifarth W, Leib-Mosch C, Greenwood AD (2006). Expression profiles of endogenous retroviruses in old world monkeys. J Virol.

[180] Stevenson BJ, Iseli C, Panji S, Zahn-Zabal M, Hide W, Old LJ, Simpson AJ, Jongeneel CV (2007). Rapid evolution of cancer/testis genes on the X chromosome. BMC Genomics.

[181] Strissel PL, Ruebner M, Thiel F, Wachter D, Ekici AB, Wolf F, Thieme F, Ruprecht K, Beckmann MW, Strick R (2012). Reactivation of codogenic endogenous retroviral (ERV) envelop genes in human endometrial carcinoma and prestages: emergence of new molecular targets. Oncotarget.

[182] Subramanian RP, Wildschutte JH, Russo C, Coffin JM (2011). Identification, characterization, and comparative genomic distribution of the HERV-K (HML-2) group of human endogenous retroviruses. Retrovirology.

[183] Sun C, Orozco O, Olson DL (2008). CRIPTO3, a presumed pseudogene, is expressed in cancer. Biochem Biophys Res Commun.

[184] Suo G, Han J, Wang X (2005). *Oct4* pseudogenes are transcribed in cancers. Biochem Biophys Res Commun.

[185] Sverdlov ED (2000). Retroviruses and primate evolution. Bioassays.

[186] Szpakowski S, Sun X, Lage JM, Dyer A, Rubinstein J, Kowakski D, Sasaki C, Costa J, Lizardi PM (2009). Loss of epigenetic silencing in tumors preferentially affects primate-specific retroelements. Gene.

[187] Talmage K, Boorstein WR, Vamvakopoulos NC, Gething M-J, Fiddes JC (1984). Only three of the seven human chorionic gonadotropin beta subunit genes can be expressed in the placenta. Nucl Acids Res.

[188] Tam OH, Aravin AA, Stein P, Girard A, Murchison EP, Cheloufi S, Hodges E, Anger M, Sachidanandam R, Schultz RM (2008). Pseudogene-derived small interfering RNAs regulate gene expression in mouse oocytes. Nature.

[189] Taylor JS, Raes J (2004). Duplication and divergence: the evolution of new genes and old ideas. Annu Rev Genet.

[190] Torrents D, Suyama M, Zdobnov E, Bork P (2003). A genome-wide survey of human pseudogenes. Genome Res.

[191] Tubio JMC, Li Y, Ju YS, et al. (74 names). Extensive transduction of nonrepetitive DNA mediated by L1 retrotransposition in cancer genomes. Science. 2014;345. doi: 10.1126/science. 1251343 10.1126/science.1251343 PMC4380235 25082706

[192] van Rijk AA, de Jong WW, Bloemendal H (1999). Exon shuffling mimicked in cell culture. Proc Natl Acad Sci U S A.

[193] van Rijk A, Bloemendal H (2003). Molecular mechanisms of exon shuffling: illegitimate recombination. Genetica.

[194] Van de Peer Y, Maere S, Meyer A (2009). The evolutionary significance of ancient genome duplications. Nature Rev Genet.

[195] Venables JP (2004). Aberrant and alternative splicing in cancer. Cancer Res.

[196] Venables JP, Klinck R, Koh C (2009). Cancer-associated regulation of alternative splicing. Nat Struct Mol Biol.

[197] Vinckenbosch N, Dupanloup I, Kaessmann H (2006). Evolutionary fate of retroposed gene copies in the human genome. Proc Natl Acad Sci U S A.

[198] Volff J-N (2006). Turning junk into gold: domestication of transposable elements and the creation of new genes in eukaryotes. BioEssays.

[199] Wang W, Zheng H, Yang S, Yu H, Li J, Jiang H, Su J, Yang L, Zhang J, McDermott J, Samudrala R, Wang J, Yang H, Yu J, Kristiansen K, Wong GKS, Wang J (2005). Origin and evolution of new exons in rodents. Genome Res.

[200] Wang-Johanning F, Frost AR, Johanning GL, Khazaeli MB, LoBuglio AF, Shaw DR, Strong TV (2001). Expression of human endogenous retrovirus k envelope transcripts in human breast cancer. Clin Cancer Res.

[201] Wang-Johanning F, Frost AR, Jian B, Azerou R, Lu DW, Chen DT, Johanning GL (2003). Detecting the expression of human endogenous retrovirus E envelope transcripts in human prostate adenocarcinoma. Cancer.

[202] Wang-Johanning F, Radvanyi L, Rycaj K, Plummer JB, Yan P (2008). Human endogenous retrovirus K triggers and antigen-specific immune response in breast cancer patients. Cancer Res.

[203] Wang-Johanning F, Li M, Esteva FJ, Hess KR, Yin B, Rycaj K, Plummer JB, Garza JG, Ambs S, Johanning GL (2014). Human endogenous retrovirus type K antibodies and mRNA as serum biomarkers of early-stage breast cancer. Int J Cancer.

[204] Watanabe T, Totoki Y, Toyoda A, Kaneda M, Kuramochi-Miyagawa S, Obata Y, Chiba H, Kohara Y, Kono T, Nakano T (2008). Endogenous siRNAs from naturally formed dsRNAs regulate transcripts in mouse oocytes. Nature.

[205] Weinberg RA (1980). Origins and roles of endogenous viruses. Cell.

[206] Wezel F, Pearson J, Kirkwood LA, Southgate J (2013). Differential expression of Oct4 variants and pseudogenes in normal urothelium and urothelial cancer. Amer J Pathol.

[207] Yu H-L, Zhao Z-K, Zhu F (2013). The role of human endoretroviral long terminal repeat sequences in human cancer. Int J Mol Med.

[208] Yu H, Liu T, Zhao Z, Chen Y, Zeng J, Liu S, Zhu F (2014). Mutations in 3′-long terminal repeat of HERV-W family in chromosome 7 upregulate syncytin-1 expression in urothelial cell carcinoma of the bladder through interacting with cMyb. Oncogene.

[209] Zendman AJ, Zschocke J, van Kraats AA, de Wit NJ, Kurpisz M, Weidle UH, Ruiter DJ, Weiss EH, van Muijen GN (2003). The human SPANX multigene family: genomic organization, alignment and expression in male germ cells and tumor cell lines. Gene.

[210] Zendman AJ, Ruiter DJ, Van Muijen GN (2003). Cancer/testis-associated genes: identification, expression profile, and putative function. J Cell Physiol.

[211] Zhang CL, Tada M, Kobayashi H, Nozaki M, Moriuchi T, Abe H (2000). Detection of *PTEN* nonsense mutation and *ψPTEN* expression in central nervous system high-grade astrocytic tumors by a yeast-based stop codon assay. Oncogene.

[212] Zhang J, Rosenberg HF (2002). Diversifyinf selection of the tumor-growth promoter angiogenin in primate evolution. Mol Biol Evol.

[213] Zhang J, Wang X, Li M (2006). NANOGP8 is a retrogene expressed in cancers. FEBS J.

[214] Zhang Q, Su B (2014). Evolutionary origin and human-specific expansion of a cancer/testis antigen gene family. Mol Biol Evol.

[215] Zhang YE, Long M (2014). New genes contribute to genetic and phenotypic novelties in human evolution. Curr Opin Genet Dev.

[216] Zhang ZD, Frankish A, Hunt T, Harrow J, Gerstein M (2010). Identification and analysis of unitary peudogenes: historic and contemporary gene losses in humans and other primates. Genome Biol.

[217] Zhang Z, Harrison PM, Liu Y, Gerstein M (2003). Millions of years of evolution preserved: a comprehensive catalog of the processed pseudogenes in the human genome. Genome Res.

[218] Zhao S, Yuan Q, Hao H (2011). Expression of *OCT4* pseudogenes in human tumors: lessons from glioma and breast carcinoma. J Pathol.

[219] Zheng PZ, Znang Z, Harrison PM (2005). Integrated pseudogene annotation for human chromosome 22: evidence for transcription. J Mol Biol.

[220] Zou M, Baitei EY, Alzahrani AS, Al-Mohanna F, Farid N, Meyer B, Shi Y (2009). Oncogenic activation of MAP kinase by *BRAF* pseudogene in thyroid tumors. Neoplasia.

